# A phase IV randomised, open-label pilot study to evaluate switching from protease-inhibitor based regimen to Bictegravir/Emtricitabine/Tenofovir Alafenamide single tablet regimen in Integrase inhibitor-naïve, virologically suppressed HIV-1 infected adults harbouring drug resistance mutations (PIBIK study): study protocol for a randomised trial

**DOI:** 10.1186/s12879-020-05240-y

**Published:** 2020-07-20

**Authors:** Collins C. Iwuji, Duncan Churchill, Stephen Bremner, Nicky Perry, Ye To, Debbie Lambert, Chloe Bruce, Laura Waters, Chloe Orkin, Anna Maria Geretti

**Affiliations:** 1 Department of Global Health and Infection, Brighton and Sussex Medical School, University of Sussex, Falmer, Brighton, BN1 9PX UK; 2 Brighton and Sussex University Hospitals NHS Trust, Brighton, UK; 3 Department of Primary Care and Public Health, Brighton and Sussex Medical School, University of Sussex, Brighton, UK; 4 Brighton & Sussex Clinical Trials Unit, University of Sussex, Brighton, UK; 5 The Mortimer Market Centre, Central and North West London NHS Foundation Trust, London, UK; 6 Barts Health NHS Trust, London, UK; 7 Queen Mary University of London, London, UK; 8 Institute of Infection, University of Liverpool, Liverpool, UK

**Keywords:** HIV, Antiretroviral drugs, Drug resistance, Protease inhibitor, B/F/TAF, Integrase inhibitor, Pilot, Phase IV randomised trial

## Abstract

**Background:**

Currently recommended boosted protease-inhibitor (bPI) regimens may be associated with increased risk of cardiovascular or chronic kidney diseases; in addition, boosted regimens are particularly associated with drug-drug interactions. Since both cardiovascular and renal disease, and polypharmacy, are common in ageing people with HIV, there is a need for alternative efficacious regimens. bPI-based regimens are often the treatment of choice for individuals with pre-treatment or treatment-acquired resistance but it is plausible that carefully selected HIV-positive individuals with drug resistance, who are virologically suppressed on their current bPI regimen, could maintain virological efficacy when switched to bictegravir, emtricitabine and tenofovir alafenamide (B/F/TAF) fixed dose combination (FDC).

**Methods/design:**

A phase IV, investigator-initiated, multicentre, open label pilot, randomised two-arm study to assess the safety and efficacy of switching from bPI regimen to B/F/TAF single tablet regimen in integrase inhibitor-naïve, virologically suppressed adults with HIV-1 infection harbouring drug resistance mutations. Eligible individuals will either continue on their bPI regimen or switch to B/F/TAF FDC. After 24 weeks, all participants in the bPI arm will be switched to B/F/TAF and followed for a further 24 weeks and all participants will be followed for 48 weeks. The primary efficacy endpoint is the proportion of participants with HIV-1 RNA < 50 copies/mL at week 24 using pure virologic response whilst the secondary efficacy endpoint is the proportion of participants with HIV-1 RNA < 50 copies/mL at Week 48. Other secondary outcome measures include between arm comparisons of drug resistance at virological failure, safety and tolerability and patient-reported outcome measures.

**Discussion:**

We aim to provide preliminary evidence of the efficacy of switching to B/F/TAF in patients with virological suppression on a bPI-based regimen who harbour select drug resistance mutations.

**Trial registration:**

ISRCTN 44453201, registered 19 June 2019 and EudraCT 2018–004732-30.

## Background

Boosted darunavir and atazanavir are recommended as preferred boosted protease inhibitors (bPI) in the British HIV Association treatment guidelines. However, boosted darunavir was associated with increased cardiovascular risk in a large prospective observational study [1]. Although boosted atazanavir has not been associated with increased cardiovascular risk [2], cohort studies indicate an increased risk of chronic kidney disease [3]. In clinical trials, atazanavir recipients experienced higher discontinuation rates from adverse events than darunavir [2, 4] which were driven by hyperbilirubinaemia and renal events. Ageing of people living with HIV is resulting in increasing prevalence of cardiovascular and renal diseases; in addition, polypharmacy is common due to comorbidities, pointing to an additional limitation for bPI due to their particularly high potential for drug-drug interactions [5, 6].

There is evidence from clinical trials that switching from virologically suppressive bPI-based antiretroviral therapy (ART) to regimens based on the newer strand transfer integrase inhibitors, bictegravir [7] and dolutegravir [8] is safe and efficacious. These studies however excluded individuals known to harbour HIV-1 drug resistance. It is well established that HIV-positive individuals that have either pre-treatment drug resistance (PDR) or limited drug resistance following failure of first-line ART achieve virological suppression on regimens comprising a bPI plus two nucleos(t)ide reverse transcriptase inhibitor (NRTIs) [9, 10]. In contrast, in the SWITCHMRK study, switching virologically suppressed individuals from a bPI to the integrase inhibitor raltegravir resulted in an increased risk of virological failure relative to individuals maintained on the bPI. A post-hoc analysis suggested that this effect might be mediated by prior virological failure compromising the activity of the NRTI backbone [11].

However, evidence indicates that second-generation INSTIs, including bictegravir and dolutegravir, have an improved barrier to resistance relative to first-generation compounds and may overcome both PDR and limited treatment-associated drug resistance. In the DAWNING study, a second line switch to a regimen comprising the INSTI dolutegravir plus 2 NRTIs, where at least one NRTI was predicted to be fully active based on resistance analysis at the time screening, was superior to a bPI plus 2 NRTIs at 24 weeks in patients failing first line ART [12]. DAWNING study suggests second-generation INSTI like dolutegravir and by extension bictegravir are likely to be successful in switch strategies in the presence of either PDR or treatment-acquired drug resistance, including M184V/I or thymidine analogue mutations (TAMs). A small, open label, single arm study switched 37 patients (54% on a bPI-based regimen) harbouring the lamivudine (3TC) and emtricitabine (FTC) associated mutation M184V/I to the fixed dose combination (FDC) of elvitegravir/cobiscitat/emtricitabine/tenofovir alafenamide with no virological failures observed after 12 weeks of follow up [13]. Another study investigated whether efficacy was maintained following a switch to bictegravir/emtricitabine/tenofovir alafenamide FDC (B/F/TAF) in individuals suppressed on either a PI-based regimen or the FDC of dolutegravir/abacavir/lamivudine (DTG/ABC/3TC). Amongst the 572 participants on B/F/TAF, 532 (93%) achieved virologic suppression (VL < 50 copies/mL), with missing virologic data accounting for the majority of the remaining. Of the 572 participants, 405 (71%) had baseline resistance data available determined by both historical genotypes and baseline proviral DNA sequence; 52 (13%) of whom had NRTI associated resistance mutation present. 35/36 (97%) of those patients with archived F/TAF resistance mutations maintained HIV-1 RNA suppression through week 48 [14].

It should be noted that in virologically suppressed patients it is often possible to recover proviral sequences from cellular reservoirs in peripheral blood [14]. The presence of archived drug resistance mutations identified by sequencing proviral DNA is not necessarily reflective of the full range of resistant variants that may have emerged in an individual. Furthermore, it does not necessarily correlate with an increased risk of failure as integrated provirus is often defective [15] and reactivation of a particular virus is likely to be a stochastic event. In studies that examine drug activity in the presence of archived drug resistance, duration of follow up is crucial because the likelihood that a particular latent virus carrying a certain mutation would reactivate may increase with time, and the levels of adherence to ART over time play a key modulating role. In the single arm switch study referred to earlier, drug resistance sequencing based on proviral DNA missed half of M184V/I mutation present in historical genotype [13].

Taken together these studies suggest that B/F/TAF may be effective in maintaining virological suppression in patients with historical evidence of drug resistance mutations. In light of this, we hypothesize that switching HIV-positive patients who harbour selected drug resistance mutations and are virologically suppressed on bPI regimen to B/F/TAF will maintain virological efficacy over 24 weeks.

## Methods

### Trial design

**Fig. 1:**
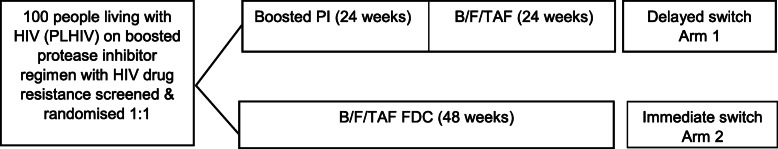
Trial design

The PIBIK trial is a phase IV, investigator-initiated, prospective, multicentre, open label pilot, randomised two arm study to assess the safety and efficacy of switching from a bPI-based regimen to B/F/TAF single tablet regimen in INSTI-naïve, virologically suppressed HIV-1 Infected adults harbouring drug resistance mutations. The allocation ratio is 1:1 (Fig. 1).

### Trial setting

Subjects will be enrolled from seven sexual health clinics in England. These are:

- Brighton and Sussex University Hospitals NHS Trust (BSUH), Brighton, UK. Other NHS Trust within the Sussex HIV network will be able to refer potentially eligible participants to BSUH for screening and if enrolled will be followed up at BSUH
- Barts Health NHS Trust, Royal London Hospital, London, UK
- The Mortimer Market Centre, Central and North West London NHS Foundation Trust
- Chelsea and Westminster Hospital NHS Foundation Trust, London, UK
- Royal Free London NHS Foundation Trust, UK
- Kings College Hospital NHS Foundation Trust, London, UK
- Guy’s and St Thomas’ NHS Foundation Trust, London, UK

### Participants

Clinical staff in the HIV department will identify potential participants by any of the following methods: review of a clinic records/database, pre-identification of patients attending for routine care, review of notes during routine clinical follow up and posters/advertisements in the clinics to inform patients of the study.

Clinical staff identifying patients will be members of the direct care team or research nurses or doctors working within the same HIV multidisciplinary team. A medically qualified doctor on the study delegation log will confirm eligibility.

Anonymised information on participants who are not randomised / registered for CONSORT (16)reporting will include the reason, if they are not eligible for trial participation, or if they are eligible but declined.

Potentially eligible participants will be invited to attend for an appointment, having been provided with a participant information sheet (PIS). Adequate time will be allowed for questions and to consider the study before agreeing to participate. The investigator or designee will provide adequate explanation of the aims, methods, objectives and potential hazards of the study. It will also be explained to the individual that they are free to refuse or withdraw from the study for any reason without detriment to their future care or treatment. The investigator can decide to withdraw a subject from the study for urgent medical reasons or repeated non- compliance with the study protocol. Once randomised, withdrawn subjects may be replaced if considered necessary by the chief investigator.

#### Inclusion criteria

- 18 years and above
- On a bPI-based ART regimen with documented HIV-1 RNA < 50 copies/mL for at least 6 months on current regimen and at screening (A switch from tenofovir disoproxil fumarate (TDF) to tenofovir alafenamide (TAF), lamivudine (3TC) to emtricitabine (FTC), or splitting co-formulated tablets to their individual component or vice versa will not be considered true regimen changes)
- Must have a historical genotype
- Eligible drug resistance mutations in historical genotype include at least one of the following:
  - o M184V/I with or without any NRTI-associated mutation (e.g. L74I/V, Y115F, K70E/G/Q/T/N/S)
  - o M184V/I alone (maximum of 20 participants with isolated M184V/I mutation with or without NNRTI mutations)
  - o Up to 2 TAMs (M41L, D67N, K70R, L210W, T215F/Y, or K219Q/E/N/R) with or without M184V/I
  - o Any of the above with or without NNRTI mutations
- No previous use of any approved or experimental INSTI
- No known INSTI mutations
- Estimated GFR ≥ 50 mL/min (Cockcroft-Gault formula)
- Have the following laboratory values at screening within 30 days prior to baseline:

- Alkaline phosphatase ≤3.0 x upper limit of normal (ULN)
- AST and ALT ≤5.0 x ULN
- Haemoglobin ≥9.0 g/dL (female) or ≥ 10.0 g/dL (male).

A single repeat of a laboratory screening test will be allowed for test results that are unexpected based on documented prior laboratory results.

- Provides written, informed consent to participate
- Is willing to comply with the protocol requirements
- If a woman and of childbearing potential and are willing to continue practicing one of the following:
  - o Must be using effective birth control methods, that is has an expected failure rate of < 1% per year and willing to continue practicing these birth control measures during the trial and for at least 30 days after the end of the trial. Effective methods include IUD, combined pill, contraceptive injection, implant, IUS, contraceptive vaginal ring, contraceptive patches etc.
  - o Must be truly abstinent from penile-vaginal intercourse from 2 weeks prior to administration of study drug, throughout the study, and for at least 30 days after the end of the trial (When this is in line with the preferred and usual lifestyle of the subject.) [Periodic abstinence (e.g., calendar, ovulation, symptothermal, post-ovulation methods), and withdrawal are not acceptable methods of contraception].
    Women who are postmenopausal for least 2 years, women with a total hysterectomy, and women who have a tubal ligation are considered of non-childbearing potential.
    If male, and sexually-active with female partners of child bearing potential, is using effective barrier contraception, and willing to continue using this during the trial and for at least 30 days after the end of the trial.

#### Exclusion criteria

- Exclusion based on drug resistance mutations include
  - o Presence of any of the following mutations: K65R/N/E
  - o Presence of multidrug resistance mutations: T69ins, Q151M with or without A62V, V75I, F77L, F116Y
  - o Three or more TAMs (M41L, D67N, K70R, L210W, T215F/Y, or K219Q/E/N/R)
- Individuals experiencing decompensated cirrhosis (e.g., ascites, encephalopathy, or variceal bleeding)
- An opportunistic illness within the 30 days prior to screening
- Active tuberculosis infection
- Have been treated with immunosuppressant therapies or chemotherapeutic agents within 3 months of study screening, or expected to receive these agents or systemic steroids during the study (e.g., corticosteroids, immunoglobulins, and other immune- or cytokine-based therapies)
- Current alcohol or substance use judged by the Investigator to potentially interfere with patients’ adherence to study procedure.
- A history of malignancy of less than 5 years or ongoing malignancy (including untreated carcinoma in-situ) other than cutaneous Kaposi’s sarcoma (KS), basal cell carcinoma, or resected, non-invasive cutaneous squamous carcinoma. Individuals with biopsy-confirmed cutaneous KS are eligible but must not have received any systemic therapy for KS within 30 days of Day 1 and are not anticipated to require systemic therapy during the study.
- Active, serious infections (other than HIV 1 infection) requiring parenteral antibiotic or antifungal therapy within 30 days prior to Day 1 (except if the parenteral therapy is for syphilis infection)
- Any other clinical condition or prior therapy that will, in the opinion of the investigator, make the patient ineligible
- Any known allergies to the excipients of B/F/TAF FDC
- Females who are pregnant (as confirmed by positive urine pregnancy test)
- Women who are breastfeeding
- Women of childbearing age not using any reliable form of contraception (e.g. intrauterine device/intrauterine system, long-acting contraceptive injection)
- Acute hepatitis in the 30 days prior to study entry, anyone with hepatitis C (HCV) who is likely to need direct acting antivirals in study
- Any concomitant medications that cannot be administered with TAF (i.e strong inducers of p-glycoprotein) or bictegravir (dofetilide, rifampins)

### Interventions

The investigational medicinal product in this trial is Biktarvy® comprising FDC of B/F/TAF. Each film-coated tablet contains bictegravir sodium equivalent to 50 mg of bictegravir, 200 mg of emtricitabine, and tenofovir alafenamide fumarate equivalent to 25 mg of tenofovir alafenamide.

Eligible individuals will either continue on their bPI regimen (arm 1) or switch to B/F/TAF FDC (arm 2). After 24 weeks, all participants in the bPI arm will be switched to B/F/TAF arm and will be followed up for a further 24 weeks whilst those immediately switched to B/F/TAF at baseline will be followed up for 48 weeks as illustrated in Fig. 1.

A participant is free to withdraw from the study at any time. In addition, the investigator may decide, for reasons of medical prudence, to withdraw a participant. If a participant discontinues study medication dosing, every attempt would be made to keep the participant in the study and continue to perform the required study-related procedures and follow-up procedures. If this is not possible or acceptable to the subject or investigator, the participant may be withdrawn from the study.

Study medication may also be discontinued in the following instances:

- If the participant withdraws his/her consent.
- If the investigator considers in the interest of the subject (i.e. intercurrent illness, unacceptable toxicity) that it is best for them to withdraw their consent.
- The participant fails to comply with the protocol requirements or fails to cooperate with the investigator.
- Pregnancy during the course of the study.

The date and reasons for the withdrawal will be clearly stated on the participant’s eCRF and source document. Every attempt should be made to arrange follow up visits for participants who are withdrawn from the trial (including where individuals fall pregnant).

Participant adherence B/F/TAF and bPI will be assessed through:

- Pill counting at each visit by a research team member and recording of the number of pills returned
- Self-report using a visual analogue scale (VAS)

Participants will bring in all pill bottles at each study visit. The total number of pills remaining at each visit will be counted and, then returned to the participant to take until the bottle is finished. The percentage of compliance for each participant will be calculated.

Participants will also be asked be asked to self-report their level of adherence at each visit using the VAS in which they would be asked to indicate their level of adherence in the previous 30 days on a scale ranging from 0 to 100% in which 0 represents no pill taken and 100 represents every single dose had been taken. Where adherence is < 80%, this will lead to likely withdrawal from the study although this will be at the discretion of the investigator.

### Outcome measures

The primary endpoint is the proportion of participants with HIV-1 RNA < 50 copies/mL at Week 24 using pure virologic response (PVR).

Pure virologic response is defined as follows:

- On study treatment
- No confirmed virologic rebound defined as:
  - o HIV RNA ≥ 50 copies/mL on 2 consecutive visits
  - o HIV-1 RNA ≥ 50 copies/mL during study followed by premature discontinuation
- Discontinuation prior to week 24 for reasons other than virologic rebound (i.e. no data in window and last HIV RNA < 50 copies/mL) are considered PVR

Secondary outcome measures include the following:

- Proportion of patients with HIV RNA < 50 copies/mL at week 48 using PVR
- Proportion of patients with HIV-1 RNA < 50 copies/mL at weeks 24 and 48 using PVR in those with any archived resistance detected in proviral DNA
- Emergence of new resistance mutations in participants with two consecutive viral load ≥50 copies/mL measured 2–3 weeks apart
- Safety and tolerability of B/F/TAF FDC in participants switching from bPI-based regimens at 48 weeks based on clinical presentation and laboratory results
- Change from baseline in patient reported outcomes at weeks 24 and 48 measured using the *HIV Symptom Distress Module (HIV-SI)* and the *Pittsburgh Sleep Quality Index (PSQI)* questionnaires.
- Change from baseline in serum lipid concentrations at weeks 24 and 48
- Change from baseline in HBA1c in blood weeks 24 and 48
- Change from baseline in weight and BMI at weeks 24 and 48

### Sample size justification

We considered a number of sample size scenarios bearing in mind the pilot nature of the study (Table 1). We will perform a futility analysis at 24 weeks when assessing the primary outcome. At 24 weeks, with 98 participants in the trial, we will have 80% power for 10% significance to conclude non-inferiority of the B/F/TAF arm assuming a non-inferiority margin of 13% and viral suppression in 90% of participants in both arms.

**Table 1:** Sample size scenarios

Confidence level	Lower limit of CI	Upper limit of CI	Sample size per arm	Total sample size
95%	80	96%	62	124
90%	80	96%	50	100
80%	80	95%	38	76

For the study to continue beyond 24 weeks, we need 90% (45/50) of the individuals randomised to the B/F/TAF arm to be suppressed with the lower limit of the confidence interval to just lie above 80%. Recruiting 50 participants per arm would achieve this at the 90% confidence level. The sample size required decreases as the level of confidence in our estimates decreases.

### Recruitment

We have allowed 12 months to recruit 100 participants over the seven sites involved in the trial. This requires recruitment of 1–2 participants per month per site which is deemed feasible. Each site has been allocated a target of 14–15 subjects. In the case of slow enrolment, additional sites will be offered participation in the study.

### Allocation and blinding

The web-based Sealed Envelope™ system will be used to allocate individuals randomly to Arm 1 and Arm 2. The statistician will provide the randomisation list. Each study site will be provided with a randomisation guide. The Sealed Envelope™ system will randomise subjects within 8 strata as shown in Table 2.

**Table 2:** Randomisation strata

Stratum	Baseline Protease-inhibitor Regimen	Use of lipid lowering therapy at study day 1	No of baseline mutations of the NRTI class^**a**^
I	Atazanavir	Yes	< 2
II	Atazanavir	Yes	≥2
III	Atazanavir	No	< 2
IV	Atazanavir	No	≥2
V	Darunavir	Yes	< 2
VI	Darunavir	Yes	≥2
VII	Darunavir	No	< 2
VIII	Darunavir	No	≥2

^a^From historical genotype report assessed during screening

The purpose of the stratification is to balance the treatment arms on important prognostic factors such as:

- The bPI used in the subject’s baseline regimen (Atazanavir or Darunavir)
- Use of lipid lowering therapy at study day 1 (yes/no)
- Number baseline mutations of the NRTI class (< 2 vs. ≥2)

Investigators randomise patients by completing an on-screen form with patient details, stratification factors, inclusion and exclusion criteria. Investigators are immediately shown the treatment allocation. Trial managers have real-time access to recruitment statistics and are notified by email of every new randomisation. The randomisation application conforms to the requirements of FDA 21 CFR part 11, Electronic Records; Electronic Signatures and ICH GCP. No-one can delete records from the randomisation database, so that all randomisations have to be accounted for. Audit log files detailing all activity on the randomisation system are available to the trial manager. Neither the investigators nor the participants will be blinded to the treatment allocation.

### Data collection

The presence of resistance mutations will be determined using historical genotype results obtained in local laboratories in those eligible according to the inclusion and exclusion criteria. We would obtain more information on resistance mutations by sequencing cell-associated HIV-1 DNA in peripheral blood mononuclear cells prior to commencing B/F/TAF. The results of proviral DNA sequence will not be available in real time and will not be used to inform treatment decisions but will further the understanding of the clinical importance of archived resistance mutations, if any, in individuals developing virological failure.

The baseline visit will not exceed 30 days after the screening visit. Follow up of participants will continue until all participants have accrued 48 weeks from their baseline visits. Individuals who have completed Week 48 visit will be followed up 30 days post cessation of trial treatment via a telephone call or a standard of care clinical appointment for performance of the following assessments; adverse events and symptoms review, HIV associated conditions, concomitant medications and for women of child bearing potential (WOCBP), confirmation that contraception has been used in the previous 30 days.

Study procedures, screening, randomisation and safety monitoring will be according to attached visit schedule in Table 3.

**Table 3:** Trial procedures and Timelines

Weeks	Screening (− 30 days)	Baseline (FV)	4	12	24 (FV)	28	36	48 (FV)	Early termination visit (FV)	30 day post treatment follow up *(telephone call or standard of care clinic visit)*
**Informed consent**	X									
**Demographic data and medical history including full ART history**	X									
**Eligibility criteria**	X	X								
**Review of historical drug resistance tests**	X									
**Adverse events**		X	X	X	X	X	X	X	X	X
**Concomitant medications**	X	X	X	X	X	X	X	X	X	X
**HIV-SI**		X			X			X	X	
**PSQI**		X			X			X	X	
**HIV associated conditions**	X	X	X	X	X	X	X	X	X	X
**Randomisation**		X								
**e-Case Report Forms**	X	X	X	X	X	X	X	X	X	X
**Vital signs (Blood Pressure, Pulse, temperature, weight)**	X	X	X		X	X		X	X	
**Complete/**^***a***^***symptom directed physical examination***	X	X^a^	X^a^	X^a^	X^a^	X^a^	X^a^	X^a^	X^a^	
**Height**	X									
**Weight, BMI & waist circumference**	X	X	X		X	X		X	X	
**12 Lead ECG**	X				X			X	X	
**Urinalysis**	X	X	X		X	X		X	X	
**Urine Pregnancy Test**	X	X	X		X	X		X	X	
**CD4/CD8 T cell count**		X			X			X	X	
**HIV-1 RNA viral load (& resistance testing if > 50 copies/mL)**	X	X	X	X	X	X	X	X	X	
**Whole blood for Proviral DNA**		X								
**Haematology**	X	X	X		X	X		X	X	
**Urea, electrolytes, creatinine, phosphate, glucose, calcium, amylase**	X	X	X		X	X		X	X	
**Estimated GFR**	X	X	X		X	X		X	X	
**UPCR**	X	X	X		X	X		X	X	
**HBV & HCV serology**	X									
**Liver function tests**	X	X	X		X	X		X	X	
**Lipids (Fasting Cholesterol, LDL, HDL, Triglycerides)**		X			X			X	X	
**HbA1c**		X			X			X	X	
**Pill count adherence**		X	X	X	X	X	X	X	X	
**Self-reported adherence**		X	X	X	X	X	X	X	X	
**Study drug dispensation**		X	X	X	X	X	X			
**Study drug accountability**			X	X	X	X	X	X	X	

*FV* Fasting visit, *HIV-SI* HIV symptom distress module, *PSQI* Pittsburgh Sleep Quality Index, *BMI* Body mass index, *UPCR* urine protein creatinine ratio, *HBV* Hepatitis B virus, *HCV* Hepatitis C virus, *LDL* Low density lipoprotein, *HDL* High density lipoprotein

Individuals with virological failure defined as a rebound in HIV-1 RNA ≥ 50 copies/mL, which is subsequently confirmed at the following scheduled or unscheduled visit. Following the initial detection of virological rebound, subjects will be asked to return to the clinic for a scheduled or unscheduled blood draw (2 to 3 weeks after the date of the first measured rebound) for repeat viral load testing. If virological rebound is confirmed and the HIV-1 RNA is ≥200 copies/mL, the blood sample from the confirmation visit will be the primary sample used for HIV-1 genotypic testing. After a participant’s first post-baseline resistance test, additional testing will be conducted on a case-by-case basis. Any participant may be discontinued at the investigator’s discretion or per local treatment guidelines. If no resistance is detected from the genotype, the participant may remain on study drugs and a repeat HIV-1 RNA measurement should be performed (2 to 3 weeks after date of test with HIV-1 RNA ≥ 50 copies/mL). Investigators should carefully evaluate the benefits and risks of remaining on study drug for each individual participant and document this assessment in the on-site medical record.

Data on patient reported outcome measures will be collected using the HIV-SI and the PSQI. The HIV-SI is a validated, self-administered 20-item health-state questionnaire for use in clinical care and research amongst people living with HIV (PLHIV)in order to identify and address common and bothersome symptoms associated with HIV treatment and disease [16]. The instrument is considered to be the gold standard in contemporary HIV-symptom research [17]. Respondents will be asked about their experience with each 20 symptoms during the past 4 weeks using a 5-point Likert scale. Response options and scores are as follows: 0) I don’t have this symptom, 1) I have this symptom and it doesn’t bother me, 2) I have this symptom and it bothers me a little, 3) I have this symptom and it bothers me, 4) I have this symptom and it bothers me a lot.

The Pittsburgh Sleep Quality Index (PSQI) is a self-rated questionnaire which assesses sleep quality and disturbances over a 30-day recall period [18]. Nineteen individual items generate seven component scores: subjective sleep quality, sleep latency, sleep duration, habitual sleep efficiency, sleep disturbances, use of sleeping medication, and daytime dysfunction. It uses a Likert scale in with the following scores: 0) Not during the past month, 1) less than once a week, 2) once or twice a week, 3) Three or more times a week. The sum of scores for these seven components yields one global score (0 to 21). A total score of 5 or greater is indicative of poor sleep quality.

Participants with early study termination from whatever cause will undergo the assessments outlined in Table 3.

### Data management

A source data worksheet will be created to capture all the relevant information and will be filed as source documentation in the participants’ notes. Questionnaires and self-report VAS scores will also be completed by the patients. A source data agreement will be completed prior to recruitment commencing to ensure all parties are aware which documents constitute source data.

Data from the source will be entered onto the electronic case report form (eCRF) on the web-based MACRO™ electronic data capture system. Data entered will be checked by the Data Manager in accordance with the Data Management Plan (supplementary appendix) and queries raised to the clinical sites via MACRO when appropriate. Clinical sites will be responsible for the entry of data into the eCRF. Patient data will be entered using study number only and no patient identifiable data will be seen by the data management team.

Direct access will be granted to authorised representatives from the sponsor, host institution and the regulatory authorities when appropriate to permit trial-related monitoring, audits and inspections.

Archiving will be the responsibility of the sponsor and all documentation will be archived for 25 years, with the exception of the health records which will be kept in accordance with UK law and local policy. The sponsor named archivist will be responsible for ensuring the documentation is prepared in line with the relevant sponsor standard operating procedures.

### Statistical analysis

All randomised patients who received at least one dose of the study medication will be included in the analysis.

Summary statistics will be presented by trial arm using median with interquartile ranges for continuous variables with skewed distributions or mean with standard deviations for normally distributed variables. Categorical variables will be summarised using frequencies and proportions.

Non-inferiority for the futility analyses will be concluded if the lower bound of a two-sided 90% CI for the difference in proportions (B/F/TAF minus boosted PI) of patients with plasma HIV-1 RNA < 50 copies/mL at week 24 is greater than − 13%.

#### Primary outcome analyses

##### Primary efficacy endpoint

Proportions of individuals with HIV RNA < 50 copies/mL at 24 weeks will be estimated using PVR.

The percentage of participants with PVR for HIV-1 RNA cut-off at 50 copies/mL at Week 24 will be summarized.

Differences between trial arms will be estimated together with 95% confidence intervals.

#### Secondary outcome analysis

##### Secondary efficacy endpoint

Proportions of individuals with HIV RNA < 50 copies/mL at 48 weeks will be estimated using PVR.

The percentage of participants with PVR for HIV-1 RNA cut-off at 50 copies/mL at Week 48 will be summarized.

Differences between trial arms will be estimated, together with 95% confidence intervals. The proportion of patients with HIV-1 RNA < 50 copies/mL at weeks 24 and 48 using PVR in those with any archived resistance detected in proviral DNA will be estimated.

The proportion of patients with any emergent drug resistance following virological rebound will be estimated.

#### Safety endpoints

The analysis of the following secondary safety outcomes will be presented as the estimated difference and 95% confidence interval between arms from baseline to 24 and 48 weeks

- Mean total Cholesterol, LDL, HDL and triglycerides will be estimated.
- Mean HBA1c
- Mean weight and BMI
- Laboratory and clinical adverse events will be described and summarised using percentages.

Treatment differences for patient reported outcomes using the HIV-SI module and the PSQI will be compared by using the prevalence of symptoms reported by each method and presented with 95% confidence intervals. Consistent with prior analyses on HIV-SI [19, 20] we would dichotomise symptoms into not bothersome (scores of 0 or 1) or bothersome (scores of 2, 3 and 4). The overall bothersome symptom count at baseline will be generated by counting the number of individual symptoms scored as bothersome. Reported poor sleep quality scores on the PSQI will be summarised by the seven components as well as the global scores by arm. The global scores will be dichotomised into poor sleep quality (score of < 5) and good sleep quality (≥5) and an exact 95% binomial confidence interval presented for the difference in prevalence between arms. .

In the event of missing data, only available data will be included in the analyses and missing data will be quantified but not imputed. For missing data relating to primary and secondary efficacy endpoints, using the principle of PVR, the last known measured viral loads will be carried forward if data is missing at the 24 and 48 week time points.

### Data safety monitoring board

A three-member independent data safety monitoring board (DSMB) has been established comprising specialists in clinical infectious diseases, HIV medicine and a clinical trial statistician. The role of the DSMB will be to safeguard the interest of the trial participants, assess safety and efficacy of the intervention and to monitor the overall conduct of the trial. The DSMB should receive and review the progress and accruing data of the trial and provide advice on the conduct of the trial to the Trial Steering Committee (TSC). The DSMB would perform an interim review of the trial’s progress including updated figures on recruitment, data quality, main outcomes and safety data and will have responsibility on the decision whether to stop or continue the trial. The DSMB will meet six-monthly but the frequency of meetings may depend on recruitment rates or other trial events. Further details on the DSMB charter can be found in the supplementary appendix.

### Adverse events monitoring and reporting

Information on all adverse events (AEs), adverse reactions (ARs), serious adverse reactions (SARs), suspected unexpected serious adverse reactions (SUSARs) and serious adverse events (SAEs) will be documented in the case report forms. AEs, SAEs, ARs, SARs and SUSARs may be directly observed, reported spontaneously by the participant or by questioning the participant at each study visit. These will be followed up until they are resolved or the participant’s participation in the study ends (i.e. until the final CRF is completed for that participant). Any untoward event that may occur subsequent to the reporting period that the investigator assesses as related to the study drug medication will also be reported as an adverse event. The adverse event reporting period will be from consent until the participant’s final study visit. After informed consent, but prior to initiation of study treatment, all SAEs and adverse events related to protocol-mandated procedures would be reported on the CRFs. Following initiation of study treatment, all AEs, regardless of cause or relationship until 30 days post cessation of trial treatment would be reported on the CRFs. In addition, all serious adverse events assessed by the investigator as related to the investigational medication would continue to be followed even after participation in the study is over. Such events would be followed until resolution, or until no further change can reasonably be expected.

### Research ethics and consent

This study is registered with the International Standard Randomised Controlled Trials Number registry (44453201) and with the European Union Drug Regulating Authorities Clinical Trials Database (2018–004732-30). The main study findings will be reported in accordance the Consolidated Standards of Reporting Trials (CONSORT) statement [21]. The study received ethical approval from the Health Research Authority (19/LO/0905) and will be conducted in accordance with the Declaration of Helsinki. Written, informed consent will be sought from participants by an appropriate member of the research team identified on the delegation log and this is mandatory prior to any study procedures. Participants would be made aware that they may not continue to be prescribed B/F/TAF after the end of the trial unless they are eligible according to NHS England prescribing criteria for tenofovir alafenamide. In this situation, participants would be switched to alternative efficacious ART combination decided by the local principal investigator.

### Data protection and patient confidentiality

All investigators and trial site staff will comply with the requirements of the General Data Protection Regulation 2018 (GDPR) [22] with regards to the collection, storage, processing and disclosure of personal information and will uphold the Act’s core principles. Personal information will be collected, kept secure, and maintained. This will involve the creation of coded, depersonalised data where the participant’s identifying information is replaced by an unrelated sequence of characters, secure maintenance of the data and the linking code in separate locations using encrypted digital files within password protected folders and storage media and limiting access to the minimum number of individuals necessary for quality control, audit, and analysis. The confidentiality of data will also be preserved when the data are transmitted to sponsors and co-investigators by using only pseudonymised codes rather than personal identifiable information. Trial data will be stored for 25 years and the principal investigator at site is the data custodian.

## Discussion

It is plausible that carefully selected HIV-positive individuals with pre-treatment or treatment-acquired resistance who are virologically suppressed on their current PI-based regimen could maintain virological efficacy when switched to B/F/TAF FDC.

The hypothesis is based on a consideration of each component of B/F/TAF. TAF, like the earlier version tenofovir disoproxil fumarate (TDF), is a prodrug of tenofovir. However, TAF yields 5-fold higher intracellular concentrations of the active moiety tenofovir diphosphate (TFV-DP) in HIV target cells than TDF, despite much lower plasma drug concentration [23].. Since TAF and TDF produce the same active metabolite, they have similar resistance profiles but it could be proposed that the higher intracellular concentrations of TFV-DP yielded by TAF could be beneficial in resistant viral isolates [24]. Furthermore, the selective conversion of TAF to TFV-DP within HIV target cells and lower levels in plasma is associated with less renal and bone toxicity [25].

HIV-1 strains harbouring the M184V/I mutation, which causes high-level resistance to 3TC and FTC, display increased or restored susceptibility to tenofovir [26]. M184V/I mutants also display a loss of fitness that reduces their replication capacity and may account for the partial residual activity of 3TC in the presence of the mutation. In ART-experienced individuals who developed virological failure whilst treated with either zidovudine or stavudine, the stepwise accumulation of TAMs resulted in increasing resistance to tenofovir, with three or more TAMs being associated with markedly reduced tenofovir susceptibility [27]. This cross resistance to tenofovir is more marked for the TAM-1 pathway of mutations (M41L, L210W, and T215Y) than the TAM-2 pathway of mutations (D67N, K70R, K219/E/N/Q/R, and T215F). As a result, we have allowed a maximum of 2 TAMs when assessing eligibility for inclusion in the trial. The revertant mutations T215S/C/D/E/I/V/N/A/L which arise from viruses that once harboured T215Y/F do not directly impact NRTI susceptibility [28]. However, both in vitro and in vivo, the effect of TAMs on tenofovir is partially reversed by the presence of the M184V/I mutation [29, 30] with 3TC maintaining residual activity in viruses harbouring this mutation [31].

Bictegravir has potent in vitro activity against laboratory strains and clinical isolates of HIV-1, a higher genetic barrier to resistance development than raltegravir (RAL) and elvitegravir (EVG), and a statistically improved resistance profile compared to RAL, EVG, and DTG against a set of patient derived INSTI-resistant viral isolates [32]. In the EARNEST [33], and MOBIDIP [31] studies, bPI given with NRTI in individuals with previous virological failure and predicted limited NRTI activity due to resistance (mainly M184V/I and TAMs) achieved high rates of virological suppression. In the DAWNING study [12], DTG a high genetic barrier INSTI, demonstrated superior virological efficacy over a bPI. Hence there is a strong scientific plausibility for bictegravir demonstrating high rates of virological efficacy in the presence of a limited pattern NRTI mutations when switching patients from bPI regimen to B/F/TAF.

We do not foresee a challenge to recruiting the 100 participants required for this trial, however recruitment will be monitored closely and if sluggish, we would activate additional sites for participation in the study. Since all sites will be utilising standardised study documents and procedures, the number of study sites should not affect data quality. Furthermore, all sites will be closely monitored for compliance with the study protocol.

### Trial status

The trial started enrolling participants on 16 September 2019 and it is anticipated that enrolment will continue until September 2020.

## Supplementary information

**Additional file 1.**

## Data Availability

Not applicable.

## Abbreviations

ART: Antiretroviral therapy
ALT: Alanine Aminotransferase
AST: Aspartate Aminotransferase
bPI: Boosted Protease Inhibitor
NRTI: Nucleoside Reverse Transcriptase Inhibitor
NNRTI: Non-Nucleoside Reverse Transcriptase Inhibitor
TAM: Thymidine Analogue Mutation
3TC: Lamivudine
FTC: Emtricitabine
TDF: Tenofovir Disoproxyl Fumarate
TAF: Tenofovir Alafenamide Fumarate
TFV-DP: Tenofovir Diphosphate
DTG: Dolutegravir
BTG: Bictegravir
RAL: Raltegravir
EVG: Elvitegravir
FV: Fasting visit
HIV-SI: HIV symptom distress module
PSQI: Pittsburgh Sleep Quality Index
BMI: Body mass index
UPCR: Urine protein creatinine ratio
HBV: Hepatitis B virus
HCV: Hepatitis C virus
LDL: Low density lipoprotein
HDL: High density lipoprotein
TSC: Trial Steering Committee
DSMB: Data Safety and Monitoring Board
AE: Adverse Event
AR: Adverse Reaction
SAE: Serious Adverse Event
SAR: Serious Adverse Reaction
SUSAR: Suspected Unexpected Serious Adverse Reaction
GDPR: General Data Protection Act
VAS: Visual Analogue Scale
PIS: Participant Information Sheet
FDC: Fixed Dose Combination
INSTI: Integrase Strand Transfer Inhibitor
