# Supplementary material for: A phase IV randomised, open-label pilot study to evaluate switching from protease-inhibitor based regimen to Bictegravir/Emtricitabine/Tenofovir Alafenamide single tablet regimen in Integrase inhibitor-naïve, virologically suppressed HIV-1 infected adults harbouring drug resistance mutations (PIBIK study): study protocol for a randomised trial
Source: BMC Infect Dis. 2020 Jul 20;20:524. doi: 10.1186/s12879-020-05240-y (PMC7370264; doi:10.1186/s12879-020-05240-y)
Supplement: Supplementary file 1 — Additional file 1. [file 12879_2020_5240_MOESM1_ESM.pdf]

This document details trial-specific procedures and should be used in conjunction with the Data Management SOP 027.

DATA MANAGEMENT PLAN

Version: 1.0 **Date:** 20th November 2019

Trial Details

**Trial Name**	PIBIK
**Protocol Version & Date**	1.0 - 01 May 2019
**Design**	Randomised (1:1), open label, multicentre pilot study
**Planned Patients**	100

Study personnel

Name	Function	Email
Dr Collins Iwuji	Chief Investigator	C.Iwuji@bsms.ac.uk
Research Governance Sponsorship	University of Sussex, Sponsor	researchsponsorship@sussex.ac.uk
Ye To	Trial Manager (TM)	Y.To@bsms.ac.uk
Chloe Bruce	Data Manager (DM)	c.bruce@bsms.ac.uk
Debbie Lambert	Database Manager (DBM)	d.lambert@bsms.ac.uk
Professor Stephen Bremner	Senior Statistician	s.bremner@bsms.ac.uk

Abbreviations

AE	Adverse Event
BSCTU	Brighton & Sussex Clinical Trials Unit
CI	Chief Investigator
CRF	Case Report Form
CTU	Clinical Trials Unit
DBM	Database Manager
DM	Data Manager
DSMB	Data Safety and Monitoring Board
eCRF	Electronic Case Report Form
GCP	Good Clinical Practice
SAE	Serious Adverse Event
SOP	Standard Operating Procedure
TM	Trial Manager
TMF	Trial Master File

Contents

1.0 Endpoint Data ... 3
1.1. Primary Endpoints ... 3
1.2. Secondary Endpoints ... 3
2.0 The Database ... 4
2.1. User Access and Training ... 4
3.0 Data Sources and Tracking ... 4
4.0 Randomisation / Registration of Patients ... 4
5.0 Query Process ... 5
6.0 Data Validation and Review ... 5
6.1. Manual checks for data review report ... 5
6.2. Protocol Compliance Checks ... 7
7.0 Safety Data ... 10
8.0 Quality Control Checks ... 11
9.0 DSMB and Interim Analysis ... 11
10.0 Database Lock & Export ... 11
11.0 Data Archiving ... 11
12.0 Sign Off ... 12
Appendix - Dynamic References ... 12

1.0 Endpoint Data

1.1. Primary Endpoints

The statistician will analyse all of the outcomes below. Therefore, data management checks will focus on the below data being present in the database. Missing data will be queried.

Endpoint name	Data Field/s used for analysis		
	**Visit Name**	**Form Name**	**Field Name**
<ENDPOINT>	<MACRO VISIT>	<MACRO FORM>	<MACRO FIELD>
To estimate proportion of participants with plasma HIV-1 RNA <50 copies/mL at Week 24 using pure virologic response (PVR)	V_Week24	F_HIVRNA	<HIVVL><br>HIV viral load (copies/mL)

1.2. Secondary Endpoints

Endpoint name	Data Field/s used for analysis		
	**Visit Name**	**Form Name**	**Field Name**
<ENDPOINT>	<MACRO VISIT>	<MACRO FORM>	<MACRO FIELD>
To estimate proportion of patients with HIV-1 RNA <50 copies/mL at week 48 using PVR	V_Week48	F_HIVRNA	<HIVVL>
To estimate proportion of patients with HIV-1 RNA <50 copies/mL at weeks 24 and 48 using PVR in those with any archived resistance detected in proviral DNA	V_Baseline<br>V_Week24<br>V_Week48	F_HIVRNA	<HIVVL>
		F_ProDNA1<br>F_ProDNA2	ALL in ProDNA1+2
To evaluate the emergence of new resistance mutations in participants with two consecutive viral load ≥50 copies/mL measured 2-3 weeks apart.	All	F_HIVRNA	<HIVVL><br><HIVVLRep>
		F_HIVResist1<br>F_HIVResist2	ALL in HIVResist1+2
To determine the safety and tolerability of B/F/TAF FDC in participants switching from boosted Protease Inhibitor-based regimens over 48 weeks	All	F_DrugDis	<DACompCalc><br><DAPatAdher><br><DAMissSp><br><DDisNRea><br><DDisNReaSp>
		F_AE	<AErel>
		F_StudyMed	<StuMedType><br><StuMedChReas>
To evaluate the between arm change from baseline in patient reported outcomes at weeks 24 and 48	V_Baseline	F_Randomisation	<RandomGrp>
	V_Baseline<br>V_Week24<br>V_Week48	F_HIVSDS<br>F_PSQI	ALL questionnaire questions<br>*PSQI10 onwards optional to complete*

To estimate the between arm mean percentage change from baseline in serum lipid concentrations at weeks 24 and 48	V_Baseline	F_Randomisation	<RandomGrp>
	V_Baseline<br>V_Week24<br>V_Week48	F_Lipids	<LipidsFCh><br><LipidsHDL><br><LipidsLDL><br><LipidsTri>
To estimate the between arm mean percentage change from baseline in HBA1c at weeks 24 and 48	V_Baseline	F_Randomisation	<RandomGrp>
	V_Baseline<br>V_Week24<br>V_Week48	F_Blood	<BloodsHbA1C>
To estimate the between arm mean percentage change from baseline in weight and BMI at weeks 24 and 48	V_Baseline	F_Randomisation	<RandomGrp>
	V_Baseline<br>V_Week24<br>V_Week48	F_PhysEx	<Weight><br><BMI>

2.0 The Database

Elsevier MACRO™ will be used to capture and store the clinical data.

2.1. User Access and Training

The database has been designed with individual logins, which give different levels of access:

- Data Entry (site staff): can enter and modify data, and respond to queries
- Monitor (CTU data manager, trial manager): can view but may not modify patient data. Monitors can raise queries and perform source data verification, and can freeze and lock data.

Each user must follow the processes in the PIBIK MACRO Training Instructions[1] and must sign the appropriate training log[2] before being granted access to the live database.

Assessment of competence will be undertaken by the Data Manager (DM) or Trial Manager (TM) who will complete the PIBIK MACRO Username Log[3].

Data entry users will have access to the PIBIK MACRO Data Entry User Guide[4].

3.0 Data Sources and Tracking

Tracking of CRFs is not required as electronic data capture is being used.

4.0 Randomisation / Registration of Patients

Registration will occur when the Registration form in MACRO™ has been completed, and patient numbers will be automatically assigned.

The web-based Sealed Envelope™ system will be used to allocate individuals randomly to either Arm 1 (bPI regimen) or Arm 2 (B/F/TAF FDC). The statistician will provide the randomisation list. The randomisation will be stratified by Protease inhibitor (Boosted atazanavir / Boosted darunavir), Use of lipid lowering therapy at baseline visit (Day 1) (Yes / No) and Number of baseline resistance mutations (NRTI) (Fewer than 2 / 2 or more).

The PIBIK Randomisation Process[5] should be followed by the study team.

5.0 Query Process

The processes for raising and tracking queries on the database are outlined in CTU SOP 027 Data Management.

6.0 Data Validation and Review

The first data review will be performed after the first patient has their first visit data entered, then reviews will take place at least every three months, dependent on recruitment rate. Once these checks are performed, a report will be produced (see Data Management SOP 027) that will be filed in the TMF. Print outs from reports/queries run will also be filed.

The DM will ensure that the checks are being performed to the timelines stated above, and will set up a Participant Review Status spreadsheet[6] to keep track of the status of the data, i.e. incomplete, complete, reviewed, frozen, and locked.

6.1. Manual checks for data review report

Check #	Check description	MACRO Visit	MACRO Page	Question(s)	Discrepancy / Process	Action to take
R1	Check for missing data	ALL	ALL	ALL	Run missing data report in MACRO DE Module	Issue data query in MACRO
R2	Check for data with warnings next to them	ALL	ALL	ALL	Run data report in MACRO DE Module filter for overridden warning status	Issue data query in MACRO
R3	Check for overridden warnings	ALL	ALL	ALL	Run data report in MACRO DE Module filter for warning status	Issue data query in MACRO
R4	Check randomisation arm correctly entered	Baseline	<F_Randomisation>	<RandomGrp>	Check the database matches the Randomisation in Sealed Envelope (list from website, or emailed to bsctu@bsms.ac.uk)	Issue data query in MACRO and/or with site
R5	Check that correct medications are reported depending on the randomisation	Baseline	<F_Randomisation>	<RandomGrp>	Check that reported medications taken and dispensed match up with randomisation arms. Check that discrepancies are properly documented on MACRO.	Issue data query in MACRO
		ALL	F_StudyMeds	<StuMedType> = 1 (bPI regimen) from Baseline for Arm 1<br><br><StuMedType> = 2 (B/F/TAF) from Baseline for Arm 2		
		ALL	F_DrugDis	<DDTabType> = 1 if Arm 2<br><DDTabType> = 2 if Arm 1		
R6	**Arm 1 only:** Check that pts are recorded as having stopped bPI and switched to Biktarvy at week 24	Baseline	<F_Randomisation>	<RandomGrp> = 1	Check that medications are switched as expected and reported accordingly on dispensation and study meds pages. Check that discrepancies are properly documented on MACRO.	Issue data query in MACRO
		Ongoing	F_StudyMeds	<StuMedType> = 1 (bPI regimen) from baseline, then<br><StuMedType> = 2 (B/F/TAF) from week 24.<br><StuMedChReas> = 7		
		V_Week24	F_DrugDis	<DDTabType> = 1		

Check #	Check description	MACRO Visit	MACRO Page	Question(s)	Discrepancy / Process	Action to take
R7	If study meds change or stop, check there is a reason recorded – AE/study med/withdrawal	ALL	F_DrugDis	<DDisNRea> =2 or 3	Check that drug dispensation / study medications / withdrawal reason (side effects) / AE (related to study drugs) match up.	Issue data query in MACRO
		Ongoing	F_StudyMed	<StuMedChReas> = 1 or 2		
		Ongoing	<F_Withdraw	<WDReason> = 3		
		Ongoing	F_AE	<AErel> = 1		
R8	For each conmed indication, check if condition is listed as medical history or AE treatment, and vice-versa	Ongoing	F_ConMed	<MedIndication>	Check conmed indications against medical history and AEs.	Issue data query in MACRO
		Ongoing	F_AE	<AETERM><br><Aetreat> = 1		
		Screening	F_Demog	<MHCondition>		
R9	If pt is reported as pregnant, reconcile database and pregnancy form	ALL	F_Urine	<BetaHCGRes> = 1	Check bsctusafety@bsms.ac.uk for pregnancy notification form, check database for positive pregnancy test, drug not dispensed due to pregnancy, or WD reason as pregnancy	Issue data query in MACRO and/or contact site
		ALL	F_DrugDis	<DDisNRea> = 5		
		Ongoing	F_Withdraw	<WDReason> = 7		
R10	If HIV viral load is raised, check for repeat tests, and if still raised, check for repeated genotypic testing	ALL	F_HIVRNA	<HIVVL> = > 50<br><HIVVLRep> = >200	Check repeat VL and genotypic testing is done if required. Check dates of tests are within range and matching (VL repeat 2-3 weeks later, and genotypic testing on that sample)	Issue data query in MACRO
		V_HIVRes	F_HIVResist1<br>F_HIVResist2	ALL questions		
R11	If new resistance detected, check outcome, if withdrawn or switched, check relevant pages complete	V_HIVRes	F_HIVResist1<br>F_HIVResist2	<HIVOutcome> = 2 (withdrawn)<br><br><HIVOutcome> = 3 (switched to new regimen)	Check outcome of resistance testing forms and if withdrawn and/or stopped meds, check these pages match up.	Issue data query in MACRO and/or contact site
		Ongoing	F_Withdraw<br>F_StudyMed	All		
R12	If participant withdrawn, check Withdrawal page completed	All	F_DrugDis	<DDisNRea> = 4<br><DDFUp> = 0	If any withdrawal reported, check Withdrawal page completed and matches other information	Issue data query in MACRO
		V_HIVRes	F_HIVResist1<br>F_HIVResist2	<HIVOutcome> = 2		
		Ongoing	F_StudyMed	<StuMedChReas> = 3		
		Ongoing	F_Withdraw	<WithdrawYN> = 1<br><WDReason>		
R13	Check any Notes and Comments for important information	ALL	ALL	ALL	If action required such as changing data point to reflect comment, raise query.	Issue data query in MACRO

6.2. Protocol Compliance Checks

Protocol deviations will be recorded on the protocol deviation log[7] by the TM and provided to the trial oversight committees as required prior to meetings, as outlined in SOP 034 Trial Oversight Committees.

Check #	Check description	MACRO Visit	MACRO Page	Question(s)	Discrepancy and Action to take
	**Inclusion criteria (all have warnings that trigger if answer=No)**				
IC1	Incl1: 18 years and above	V_Screening	F_Registration<br><br>F_Eligibility	<ptdob><br><br><incl1>	If warning triggered, query with site
IC2	Incl2: On a bPI-based ART regimen with documented HIV-1 RNA <50 copies/mL for at least 6 months on current regimen and at screening	V_Screening	F_Eligibility<br><br><br>F_HIVRNA	<incl2><br><inc2a> = not blank<br><br><HIVVL>, <HIVRNAdate>	If warning triggered, query with site<br><br>If <inc2a> blank, query with site
IC3	Incl3: Must have a historical genotype	V_Screening	F_ARVRes1<br>F_ARVRes2<br><br>F_Eligibility	ALL questions in ARVRes1 + 2 should be complete<br><br><incl3>	If warning triggered, query with site<br><br>If missing answers, query with site
IC4	Incl4: Eligible drug resistance mutations in historical genotype include the following:<br>a. M184V/I with or without any nucleoside analogue mutation (e.g. L74I/V, Y115F, K70E/G/Q/T/N/S)<br>b. M184V/I alone<br>c. Up to 2 TAMs (M41L, D67N, K70R, L210W, T215F/Y, or K219Q/E/N/R) with or without M184V/I<br>d. Any of the above with or without NNRTI mutations	V_Screening	F_ARVRes1<br>F_ARVRes2<br><br><br><br><br>F_Eligibility	ALL<br><br><br><br><br><br><incl4>	Collins to check all historic genotype reports prior to randomisation of patients.<br><br>If warning triggered, query with site
IC5	Incl5: No previous use of any approved or experimental integrase strand transfer inhibitor (INSTI)	V_Screening	F_ARTHist<br><br><br><br>F_Eligibility	<ARTDrug>= Dolutegravir, Elvitegravir, Raltegravir, Bictegravir, Cabotegravir,<br><br><incl5>	If any are present, query with site<br><br><br>If warning triggered, query with site
IC6	Incl6: No known INSTI mutations	V_Screening	F_ARVRes2<br><br><br>F_Eligibility	<ARVInt> if Yes, patient ineligible.<br><br><incl6>	If warning triggered, query with site
IC7	Incl7: Estimated GFR ≥ 50 mL/min (Cockcroft-Gault formula)	V_Screening	F_Blood<br><br><br>F_Eligibility	<BloodseGFR> if <50, ineligible<br><br><incl7>	If warning triggered, query with site
IC8	Incl8: Have the following laboratory values at screening within 30 days prior to baseline<br>a. Alkaline phosphatase ≤ 3.0 x upper limit of normal (ULN)<br>b. AST and ALT ≤ 5.0 x ULN<br>c. Hemoglobin ≥9.0 g/dL (female) or ≥10.0 g/dL (male)<br>**Note:** A single repeat of a screening test will be allowed for results that are unexpected based on documented prior results.	V_Screening	F_Blood<br><br>F_Demog<br><br><br><br><br><br><br><br>F_Eligibility	<BloodsALP> = <387<br><BloodsAST> = <200<br><BloodsALT> = <205<br><br><BloodsHaem> = >10 and <Dmgender> = 1 (male)<br><br><BloodsHaem> = >9 and <Dmgender> = 2 (female)<br><br><incl8>	If warning triggered, query with site

Check #	Check description	MACRO Visit	MACRO Page	Question(s)	Discrepancy and Action to take
IC9	Incl9: Provides written, informed consent to participate	V_Screening	F_Eligibility	<incl9>	Cannot be checked on MACRO, would need to be confirmed at monitoring visits
IC10	Incl10: Is willing to comply with the protocol requirements	V_Screening	F_Eligibility	<incl10>	Cannot be checked on MACRO, would need to be confirmed at monitoring visits
IC11	Incl11: If female and of child bearing potential: *(shortened)*<br>a. is using effective birth control methods…<br>b. Must be truly abstinent…<br>**Note:** …postmenopausal, total hysterectomy, tubal ligation - non-childbearing potential.	V_Screening	F_Eligibility	<incl11>	Cannot be checked on MACRO, would need to be confirmed at monitoring visits
IC12	Incl12: If male, and sexually-active with female partners of child bearing potential, is using effective barrier contraception, and willing to continue using this during the trial and for at least 30 days after the end of the trial.	V_Screening	F_Eligibility	<incl12>	Cannot be checked on MACRO, would need to be confirmed at monitoring visits
	**Exclusion criteria (all have warnings that trigger if answer=Yes)**				
EC1	Excl1: Exclusion under drug resistance mutations include:<br>a. Presence of any of the following mutations: K65R/N/E<br>b. Presence of multidrug resistance mutations: T69ins, Q151M with or without A62V, V75I, F77L, F116Y<br>c. three or more TAMs (M41L, D67N, K70R, L210W, T215F/Y, or K219Q/E/N/R)	V_Screening	F_ARVRes1<br><br>F_Eligibility	<ARVK65R><br><ARVK65N><br><ARVK65E><br><ARVT69Ins><br><ARVQ151M><br><br>All TAMS<br><br><excl1>	For named mutations, warning will trigger if present at screening, query with site<br><br>Collins to check all historic genotype reports prior to randomisation of patients.<br><br>If warning triggered, query with site
EC2	Excl2: Individuals experiencing decompensated cirrhosis (e.g., ascites, encephalopathy, or variceal bleeding)	V_Screening	F_Demog<br><br>F_Eligibility	<MHCondition> (decompensated cirrhosis etc), <MHStart> Ongoing=Yes<br><br><excl2>	Manual check, if any present, query with site.<br><br>If warning triggered, query with site
EC3	Excl3: An opportunistic illness within the 30 days prior to screening	V_Screening	F_Eligibility	<excl3>	If warning triggered, query with site
EC4	Excl4: Active tuberculosis infection	V_Screening	F_Demog,<br><br>F_Eligibility	<MHCondition> (tuberculosis), <MHStart> Ongoing=Yes<br><br><excl4>	Manual check, if present, query with site.<br><br>If warning triggered, query with site

Check #	Check description	MACRO Visit	MACRO Page	Question(s)	Discrepancy and Action to take
EC5	Excl5: Have been treated with immunosuppressant therapies or chemotherapeutic agents within 3 months of study screening, or expected to receive these agents or systemic steroids during the study (e.g., corticosteroids, immunoglobulins, and other immune- or cytokine based therapies)	V_Screening	F_Demog<br><br>F_Eligibility	<DMnonARVs><br><br><excl5>	Cannot be checked on MACRO, would need to be confirmed at monitoring visits
EC6	Excl6: Current alcohol or substance use judged by the Investigator to potentially interfere with subjects' adherence to study procedure	V_Screening	F_Eligibility	<excl6>	Cannot be checked on MACRO, would need to be confirmed at monitoring visits
EC7	Excl7: A history of or ongoing malignancy (including untreated carcinoma in-situ) other than cutaneous Kaposi's sarcoma (KS), basal cell carcinoma, or resected, non-invasive cutaneous squamous carcinoma. Individuals with biopsy-confirmed cutaneous KS are eligible, but must not have received any systemic therapy for KS within 30 days of Day 1 and are not anticipated to require systemic therapy during the study	V_Screening	F_Demog,<br><br>F_Eligibility,	<MHCondition>, Malignancies, carcinomas etc<br>(Kaposi's sarcoma, BCC, SC are accepted)<br><br><DMnonARVs><br><br><excl7>	Manual check, if present, query with site.<br><br>If warning triggered, query with site
EC8	Excl8: Active, serious infections (other than HIV 1 infection) requiring parenteral antibiotic or antifungal therapy within 30 days prior to Day 1 (except if the parenteral therapy is for syphilis infection)	V_Screening	F_Demog<br><br>F_Eligibility	<MHCondition>, <MHDiagYr>, (Baseline visit date -30 days)<br><DMnonARVs> - antibiotics or antifungals<br><br><excl8>	Manual check, if present, query with site.<br><br>If warning triggered, query with site
		V_Baseline	F_AE<br><br>F_Conmed	<AESOC> = 11 (infections and infestations)<br><AESTDAT> = before Baseline date<br><br><MedName> - antibiotic or antifungal<br><MedStart> = before Baseline date	Manual check, if present, query with site.
EC9	Excl9: Any other clinical condition or prior therapy that will, in the opinion of the investigator, make the subject ineligible.	V_Screening	F_Eligibility	<excl9>	If warning triggered, query with site
EC10	Excl10: Any known allergies to the excipients of B/F/TAF FDC	V_Screening	F_Demog<br><br>F_Eligibility	<DMAllerg> = any of: Microcrystalline cellulose, Croscarmellose sodium, Magnesium stearate, Polyvinyl alcohol, Titanium dioxide (E171), Macrogol (polyethylene glycol), Talc, Iron oxide red (E172), Iron oxide black (E172)<br><br><excl10>	Manual check, if present, query with site.<br><br>If warning triggered, query with site

Check #	Check description	MACRO Visit	MACRO Page	Question(s)	Discrepancy and Action to take
EC11	Excl11: Females who are pregnant (as confirmed by positive urine pregnancy test)	V_Screening	F_Urine<br><br>F_Eligibility	<BetaHCGRes>=1 (positive)<br><br><excl11>	If warning triggered, query with site
EC12	Excl12: Females who are breastfeeding	V_Screening	F_Eligibility	<excl12>	Cannot be checked on MACRO, would need to be confirmed at monitoring visits
EC13	Excl13: Women of child bearing age not using any reliable form of contraception (e.g. intrauterine device/intrauterine system, long-acting contraceptive injection)	V_Screening	F_Eligibility	<excl13>	Cannot be checked on MACRO, would need to be confirmed at monitoring visits
EC14	Excl14: Acute hepatitis in the 30 days prior to study entry, anyone with HCV who is likely to need direct acting antivirals in study	V_Screening	F_HBVHCV<br><br>F_Eligibility	<HBsAg>=1 positive, warning<br><HCVrna>=1 detectable, warning<br><br><excl14>>	If warning triggered, query with site
EC15	Excl15: Any concomitant medications that cannot be administered with TAF (i.e. strong inducers of p-glycoprotein) or bictegravir (dofetilide, rifampins)	Ongoing	F_ConMed<br><br>F_Eligibility	<MedName> look for:<br>Antiarrhythmics: dofetilide<br>Anticonvulsants: Carbamazepine, Oxcarbazepine, Phenobarbital, phenytoin<br>Antimycobacterials: rifabutin, rifampin, rifapentin<br>Herbal Products: St. John's wort<br>Medications or oral supplements containing polyvalent cations (e.g., Mg, Al, Ca, Fe)<br>Calcium or iron supplements, Cation-containing antacids or laxatives, Sucralfate Buffered medications<br>Metformin<br><br><excl15>	Manual check<br><br>If warning triggered, query with site

7.0 Safety Data

It has been agreed with the sponsor that lab abnormalities judged not to be of clinical significance do not need reporting as adverse events/reactions. Adverse events will be recorded and reported in accordance with BSCTU Safety Reporting SOP 018.

Non-serious Adverse Events that are not related to the IMP do not require documentation in the CRF.

Both Serious Adverse Events and non-serious Adverse Events will be recorded in the MACRO™ database Adverse Events form. Adverse Events will be collected from the time of consent. The relationship of adverse events to either bPI, Biktarvy, or not related, must be determined by the investigator.

An Initial SAE Report form should be completed by the local investigators and emailed to the Brighton and Sussex Clinical Trials Unit at BSCTUsafety@bsms.ac.uk within 24 hours of the site becoming aware of the SAE occurrence. The Brighton and Sussex CTU will communicate with the CI and Sponsor.

Pre-existing conditions and planned admissions do not need reporting as SAEs.

At least every 6 months during the study, the two databases must be reconciled to ensure all information has been appropriately captured and reported to the regulatory bodies as applicable.

The independent Data Safety Monitoring Board (DSMB) will meet to address any safety concerns, review any ethical issues raised, and monitor adverse events. Prior to this DSMB meeting, reconciliation can be undertaken by the DM using the SAE Reconciliation Report[8].

The TM will provide the sponsor's list of SAEs to the DM who will check that the information matches that in the MACRO™ database.

Discrepancies and any action taken will be recorded on the reconciliation report.

	SAE Reconciliation Checks				
No.	Check details	Data points	Process	Action to take	When to run
AE1	Check for cases found in SAE log but not in MACRO	Adverse Event term, severity grade, start and stop dates, causality, outcome, action taken, treatment given.	Compare with sponsor SAE log	Raise discrepancy on MACRO	<6-monthly and/or prior to DSMB>
AE2	Check for cases found in MACRO but not in the SAE log	As before	Compare with sponsor SAE log	Raise discrepancy on MACRO and alert TM	<6-monthly and/or prior to DSMB>
AE3	Compare data points between SAE reports and eCRF AE page	As before	Compare with sponsor SAE log	Raise discrepancy on MACRO	<6-monthly and/or prior to DSMB>

8.0 Quality Control Checks

No data entry will be performed at the CTU, therefore quality control checks of data entry will not be required.

Automated edit-checks will raise warnings at the time of data entry. The data entry user will have the option to check and amend the data that is being entered or they can override the data warning, provided the data is correct per the source documents. These overridden warnings will be checked by the DM or TM (see section 6.1).

9.0 DSMB and Interim Analysis

The Data Safety Monitoring Board will review results on an interim basis.

10.0 Database Lock & Export

The database lock and unlock process will follow the SOP 036 Database Lock & Unlock. Users with Monitor level access can freeze and lock the data in MACRO™.

11.0 Data Archiving

When the study is finished and closed, a data extract of the entire study (locked datasets) will be exported by the DBM and archived on the CTU servers. This can then be used for onward dissemination by the study

team. The data extract and study essential documents will be archived for at least 25 years after the end of the study.

All data management documentation will be archived alongside the Trial Master File as detailed in the Archiving SOP 017.

12.0 Sign Off

If updates are required once a trial is live, the DM should update the Data Management Plan if necessary, change the version and update the version history below. This should be signed off by the DM, TM and Statistician.

It is the responsibility of the DM to ensure all CTU staff entering data for the trial read and understand the updates.

Data Management Plan Version:	1.0

Name	Position	Signature	Date (DD/MM/YYYY)
Chloe Bruce	Document Author - Data Manager (DM)	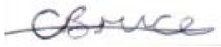	20/11/2019
Debbie Lambert	Database Manager (DBM)	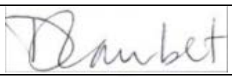	25/11/2019
Ye To	Trial Manager (TM)	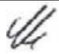	22/11/2019
Stephen Bremner	Senior Statistician	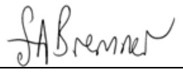	26/11/2019

DMP Version History

When there are changes to the Data Management Plan the sections should be detailed and a summary of changes provided.

DMP Version Number	Section updated	Summary of updates
1.0	All	First version.

Appendix - Dynamic References

[1] PIBIK MACRO Training Instructions, A user guide for site staff for completing and documenting training for MACRO, DM, TMF 21.05

[2] PIBIK MACRO training log, Training of study staff, DM, TMF 21.05

[3] PIBIK MACRO Username Log, A record of users and their access privileges, DM, TMF 21.05

[4] PIBIK MACRO Data Entry User Guide, Database instructions for site staff, DM, TMF 21.05

[5] PIBIK Randomisation Instructions, Guide for process of randomising patients, TM, TMF 15.01

[6] PIBIK Participant Review Status spreadsheet, Log of data review status and issues, DM, TMF 21.06

[7] PIBIK Deviation Log, Log of protocol deviations, TM, TMF 11.3

[8] PIBIK SAE Reconciliation Report, List of checks to perform and of any actions taken with discrepancies, DM, TMF

PIBIK Study

Data Safety and Monitoring Board (DSMB) Charter

Version 2.0 dated 22 Nov 2019

Study/Trial Full Title: A Phase IV Randomised, Open-Label Pilot Study to Evaluate Switching from Protease-Inhibitor based regimen to Bictegravir/Emtricitabine/Tenofovir Alafenamide Single Tablet Regimen in Integrase Inhibitor-naïve, virologically suppressed HIV-1 infected adults harbouring drug resistance mutations

Protocol short title: PIBIK study

**Chief Investigator:**	**Dr Collins Iwuji**
**Sponsor:**	**University of Sussex**
**Sponsor Reference/ IRAS ID:**	**257865**
**EudraCT Number**	**2018-004732-30**
**REC number**	**19/LO/0905**
**ISRCTN Number**	**44453201**

Authorised by:

Name: Dr Collins Iwuji
Role: Chief Investigator
Signature: [signature]
Date: 26 Nov 2019

Prepared by

Name: Ye To
Role: Clinical Trial Manager
Signature: [signature]
Date: 22 Nov 2019

Introduction

The purpose of this document is to describe the roles and responsibilities of the Data Safety and Monitoring Board for the PIBIK trial, including timing of meetings, methods of providing information to and from the DSMB, frequency and format of meetings, statistical issues and relationships with other committees.

To safeguard the interests of the trial's participants, to assess the safety and efficacy of the interventions during the trial and to monitor the trial's overall conduct.

The DSMB should receive and review the progress and accruing data of this trial and provide advice on the conduct of the trial to the Trial Steering Committee (TSC).

The DSMB should perform an interim review of the trial's progress including updated figures on recruitment, data quality, main outcomes and safety data.

DSMB members will agree to this document to register their assent. This can be done by signing the Agreement and Competing Interests form (Appendix 1).

The DSMB is an advisory committee and will make recommendations to the TSC chair

The frequency of meetings may depend on recruitment rates or other trial events but the DSMB will meet 6 monthly once recruitment has begun.

The DSMB reports its recommendations in writing to the Trial Steering Committee. This should be copied to the trial statistician (or trial manager) and if possible should be sent via the BSCTU in time for consideration at a TSC meeting. If the trial is to continue largely unchanged then it is often useful for the report from the DSMB to include a summary paragraph suitable for trial promotion purposes.

This will be a letter to the Trial Steering Committee within 2 weeks of the meeting. A copy of this is filed in the TMF located in the CTU office.

DSMB members should be named and their affiliations listed in the main report, unless they explicitly request otherwise. A brief summary of the timings and conclusions of DSMB meetings should be included in the body of this paper.

CONTENT	DETAIL
**Introduction**	
Name of Trial	**PIBIK Study:** A Phase IV Randomised, Open-Label Pilot Study to Evaluate Switching from Protease-Inhibitor based regimen to Bictegravir/Emtricitabine/Tenofovir Alafenamide Single Tablet Regimen in Integrase Inhibitor-naïve, virologically suppressed HIV-1 infected adults harbouring drug resistance mutations
Objectives of trial, including interventions being investigated	**Hypothesis**<br>Switching patients with HIV who harbour selected drug resistance associated mutations and are virologically suppressed on a bPI-based ART regimen to B/F/TAF FDC will maintain virological efficacy over 24 weeks.<br><br>**Primary objectives**<br>• To determine proportion of participants with plasma HIV-1 RNA <50 copies/mL at Week 24 post-switch to B/F/TAF FDC using pure virologic response (PVR)<br><br>**Secondary objectives**<br>• To estimate proportion of patients with HIV-1 RNA <50 copies/mL at week 48 using PVR<br>• To estimate proportion of patients with HIV-1 RNA <50 copies/mL at weeks 24 and 48 using PVR in those with any archived resistance detected in proviral DNA<br>• To evaluate the emergence of new resistance mutations in participants with two consecutive viral load ≥50 copies/mL measured 2-3 weeks apart.<br>• To determine the safety and tolerability of B/F/TAF FDC in participants switching from Bpi-based regimens over 48 weeks<br>• To evaluate the between arm change from baseline in patient reported outcomes at weeks 24 and 48<br>• To estimate the between arm mean percentage change from baseline in serum lipid concentrations at weeks 24 and 48<br>• To estimate the between arm mean percentage change from baseline in HBA1c at weeks 24 and 48<br>• To estimate the between arm mean percentage change from baseline in weight and BMI at weeks 24 and 48<br><br>Please refer to trial design in Figure 1

CONTENT	DETAIL
Outline of scope of charter	The purpose of this document is to describe the roles and responsibilities of the Data Safety and Monitoring Board for the PIBIK trial, including timing of meetings, methods of providing information to and from the DSMB, frequency and format of meetings, statistical issues and relationships with other committees.
**Roles and responsibilities**	
A broad statement of the aims of the committee	To safeguard the interests of the trial's participants, to assess the safety and potential efficacy of the interventions during the trial and to monitor the trial's overall conduct.
Terms of reference	The DSMB should receive and review the progress and accruing data of this trial and provide advice on the conduct of the trial to the Trial Steering Group.<br>The DSMB should inform the Chair of the Trial Steering Group if, in their view:<br>• the results are likely to convince a broad range of clinicians, including those supporting the trial and the general clinical community, that on balance one trial arm is clearly indicated or contraindicated for all participants or a particular category of participants, and there was a reasonable expectation that this new evidence would materially influence patient management;<br>or<br>• it becomes evident that no clear outcome would be obtained.
Specific roles of DSMB	The DSMB should perform an interim review of the trial's progress including updated figures on recruitment, data quality, main outcomes and safety data.<br><br>Specifically:<br>• monitoring recruitment figures and losses to follow up<br>• monitoring compliance with the protocol by participants and investigators<br>• monitoring evidence for treatment harm (e.g. AEs, SAEs)<br>• recommending whether the trial continues to recruit participants or whether recruitment should be terminated<br>• monitoring compliance with previous recommendations from the DSMB
**Before, or early into the trial**	
Whether the DSMB will have input into the protocol	Before recruitment began, the trial protocol had undergone review by the funder, the sponsor, the MHRA and the research ethics committee.<br>No input was requested from DSMB members before the start of the trial

CONTENT	DETAIL
	and membership of the committee was agreed prior start of recruitment of the trial.
Whether the DSMB will meet before the start of the trial	The first DSMB meeting should take place within 6 months of the start of trial recruitment, and then every 6 months during the study, but may change dependent on the rate of recruitment or events in the trial. The DSMB will always meet before the TSC so DSMB recommendations can be feedback to the TSC.
Any specific regulatory issues	This is classified by the MHRA as a **Type B study**: Somewhat higher than the risk of standard medical care. The trial involves an authorised IMP being used outside of its licensed indication.
Whether members of the DSMB will have a contract	DSMB members will agree to this document to register their assent. This can be done by signing the PIBIK DSMB Agreement and Competing Interests form (Appendix 1).
**Composition**	
Membership and size of the DSMB	The members of the DSMB for this trial are:<br>(1) **DSMB Chair:** Professor Ravindra Gupta, rkg20@cam.ac.uk (Independent Infectious Diseases Consultant)<br>(2) Professor David Dunn, d.dunn@ucl.ac.uk (Independent Statistician)<br>(3) Dr Nicola Mackie, nicola.mackie@nhs.net (Independent HIV Consultant)
The chair, how they are chosen and the chair's role. (Likewise, if relevant, the vice-chairman)	The Chair should have previous experience of serving on DSMBs and experience of chairing meetings, and should be able to facilitate and summarise discussions. The Chair is sometimes chosen by the sponsor or the investigators running the trial and sometimes by the DSMB members themselves.
The responsibilities of the DSMB statistician	The DSMB membership will include a statistician to provide independent statistical expertise.
The responsibilities of the trial statistician	The trial statistician, Dr Stephen Bremner will produce (or oversee the production of) the report to the DSMB and will participate in DSMB meetings, guiding the DSMB through the report, participating in DSMB discussions and, on some occasions, taking notes.
The responsibilities of the CTU members of the trial team	The CTU Trial Manager will assist in the setup of the meeting and will be available to take minutes in the open session.

CONTENT	DETAIL
The responsibilities of the CI and other members of the TMG	The CI, may be asked, and should be available, to attend open sessions of the DSMB meeting. The other TMG members will not usually be expected to attend but can attend open sessions when necessary (See Organisation of DSMB Meetings).
**Relationships**	
Relationships with Principal Investigators, other trial committees (e.g. Trial Steering Committee (TSC)), sponsor and regulatory bodies	Please refer to figure 2
Clarification of whether the DSMB are advisory (make recommendations) or executive (make decisions)	The DSMB is an advisory committee and will make recommendations to the TSC chair
Any payments to DSMB members	The CTU does not expect to pay DSMB members or their employers other than reimbursement for any reasonable travel or other costs (e.g. telephone) incurred.
The need for DSMB members to disclose information about any competing interests	Competing interests should be disclosed. These are not restricted to financial matters – involvement in other trials or intellectual investment could be relevant. Although members may well be able to act objectively despite such connections, complete disclosure enhances credibility. Most competing interests are acceptable if disclosed (see Appendix 1).
**Organisation of DSMB meetings**	
Expected frequency of DSMB meetings	The frequency of meetings may depend on recruitment rates or other trial events but the DSMB will meet at least 6 monthly once recruitment has begun.
Whether meetings will be face-to-face or by teleconference	Where possible, the first meeting should ideally be face-to-face to facilitate full discussion and allow members to get to know each other. It is recommended that all subsequent meetings will be by teleconference.<br>An introductory face to face meeting is advised to take place once the study is open to recruitment.
How DSMB meetings will be organised, especially regarding open and closed sessions, including who will be present in each session	A mixture of open and closed sessions is recommended.<br>Only DSMB members and others whom they specifically invite, e.g. the trial statistician, are present in closed sessions.<br>In open sessions, all those attending the closed session are joined by the PI(s), trial manager and sometimes also representatives of the sponsor, funder, or regulator, as relevant.

CONTENT	DETAIL
**Trial documentation and procedures to ensure confidentiality and proper communication**	
Intended content of material to be available in open sessions	Open sessions: Accumulating information relating to recruitment and data quality (e.g., data return rates, treatment compliance) based on pooled data will be presented, together with, total numbers of events for the primary outcome measure and other outcome measures.
Intended content of material to be available in closed sessions	Closed sessions: In addition to all the material available in the open session, the closed session material will include efficacy and safety data by treatment group. The DSMB members do not have the right to share this information with anyone outside of the DSMB.
Will the DSMB be blinded to the treatment allocation?	Not applicable
Who will see the accumulating data and interim analysis	The people who will see the accumulating data and interim analysis are:-<br>(1) **DSMB Chair:** Professor Ravindra Gupta, rkg20@cam.ac.uk (Independent Infectious Diseases Consultant)<br>(2) Professor David Dunn, d.dunn@ucl.ac.uk (Independent Statistician)<br>(3) Dr Nicola Mackie, nicola.mackie@nhs.net (Independent HIV Consultant)<br>DSMB members do **not** have the right to share confidential information with anyone outside the DSMB, including the CI.
Who will be responsible for identifying and circulating external evidence (e.g. from other trials/ systematic reviews)	The CI and the trial team, including the trial statistician will collate any such information.
To whom the DSMB will communicate the decisions/recommendations that are reached	The DSMB reports its recommendations in writing to the Trial Steering Committee. This should be copied to the trial statistician (or trial manager) and if possible should be sent via the BSCTU in time for consideration at a TSC meeting. If the trial is to continue largely unchanged then it is often useful for the report from the DSMB to include a summary paragraph suitable for trial promotion purposes.
Whether reports to the DSMB be available before the meeting or only at/during the meeting	The DSMB will receive the report at least 2 weeks before any meetings.

CONTENT	DETAIL
What will happen to the confidential papers after the meeting	The DSMB members should destroy their reports after each meetings. Fresh copies of previous reports will be circulated with the newest report before each meeting.
**Decision making**	
What decisions/recommendations will be open to the DSMB?	The possible recommendations are numerous but could include:<br>• No action needed, trial continues as planned<br>• Early stopping due to, amongst other things, clear benefit or harm of a treatment, safety concerns, futility, slow recruitment, or external evidence<br>• Extension of recruitment or follow-up<br>• Advising on or proposing protocol changes
The role of formal statistical methods, specifically which methods will be used and whether they will be used as guidelines or rules	Not applicable
How decisions or recommendations will be reached within the DSMB	Issues to be specified can include:<br>• The decision making methods and criteria that will be adopted for guiding deliberations<br>• The process of decision making, including whether there will be voting or other formal methods of achieving consensus. The method of deliberation should not be revealed to the overseeing committee as this may reveal information about the status of the trial's data.<br>• The role of the Chair: to summarise discussions and encourage consensus; it may be best for the Chair to give their own opinion last.<br>It is recommended that every effort should be made for the DSMB to reach a unanimous decision. If the DSMB cannot achieve this, a vote may be taken, although details of the vote should not be routinely included in the report to the TSC as these may inappropriately convey information about the state of the trial data.<br>It is important that the implications (e.g. ethical, statistical, practical, and financial) for the trial be considered before any recommendation is made.

CONTENT	DETAIL
When the DSMB is quorate for decision-making	Effort should be made for all members to attend. The CTU team will try to ensure that a date is chosen to enable this. Members who cannot attend in person should be encouraged to attend by teleconference. If, at short notice, any DSMB members cannot attend at all then the DSMB may still meet if at least one statistician and one clinician, including the Chair (unless otherwise agreed), will be present. If the DSMB is considering recommending major action after such a meeting the DSMB Chair should talk with the absent members as soon after the meeting as possible to check they agree. If they do not, a further teleconference should be arranged with the full DSMB.
Can DSMB members who cannot attend the meeting input?	If the report is circulated before the meeting, DSMB members who will not be able to attend the meeting may pass comments to the DSMB Chair for consideration during the discussions.
What happens to members who do not attend meetings?	If a member does not attend a meeting, it should be ensured that the member is available for the next meeting. If a member does not attend a second meeting, they should be asked if they wish to remain part of the DSMB. If a member does not attend a third meeting, they should be replaced.
Whether different weight will be given to different endpoints (e.g. safety/efficacy)	The DSMB will review all the AEs from the study, and will discuss the safety and efficacy of the study based on these.
Any specific issues relating to the trial design that might influence the proceedings, e.g. cluster trials, equivalence trials, multi-arm trials	Not applicable.
**Reporting**	
To whom the DSMB will report their recommendations/decisions, and in what form	This will be a letter to the Trial Steering Committee within 2 weeks of the meeting. A copy of this is filed in the TMF in the CTU office.
Whether minutes of the meeting be made and, if so, by whom and where they will be kept	Minutes of the meeting will be made by the Trial Manager, (separate records may be required for open and closed sessions). The DSMB Chair should sign off any minutes or notes. Minutes will be filed in the Trial Master File, kept in the CTU.
What will be done in the instances of disagreement between the DSMB and the	If the DSMB has serious problems or concerns with the TSC decision, a meeting of these groups will be held. The information to be shown would depend upon the action proposed and the DSMB's concerns. Depending on the reason for the disagreement confidential data will often have to be

CONTENT	DETAIL
body to which they report?	revealed to all those attending such a meeting. The meeting will be chaired by a senior member of the CTU staff or an external expert who is not directly involved with the trial.
**After the trial**	
Publication of results	At the end of the trial there may be a meeting to allow the DSMB to discuss the final data with principal trial investigators/sponsors and give advice about data interpretation<br>The DSMB may wish to see a statement that the trial results will be published in a correct and timely manner.
The information about the DSMB that will be included in published trial reports	DSMB members should be named and their affiliations listed in the main report, unless they explicitly request otherwise. A brief summary of the timings and conclusions of DSMB meetings should be included in the body of this paper.
Whether the DSMB will have the opportunity to approve publications, especially with respect to reporting of any DSMB recommendation regarding termination of a trial	The DSMB may wish to be given the opportunity to read and comment on any publications before submission.
Any constraints on DSMB members divulging information about their deliberations after the trial has been published	The DSMB may discuss issues from their involvement in the trial e.g. 12 months after the primary trial results have been published, or when permission is agreed with Chief Investigator.

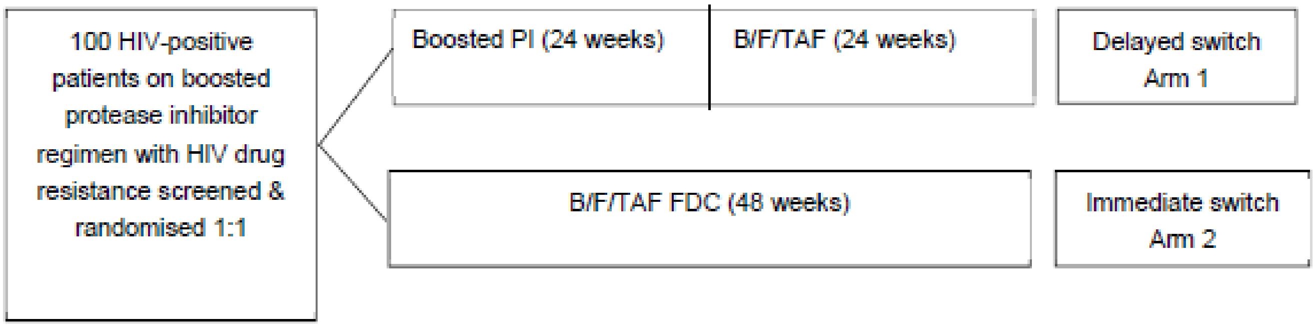

Figure 1 Trial design

Figure 2: Relationship of trial committees

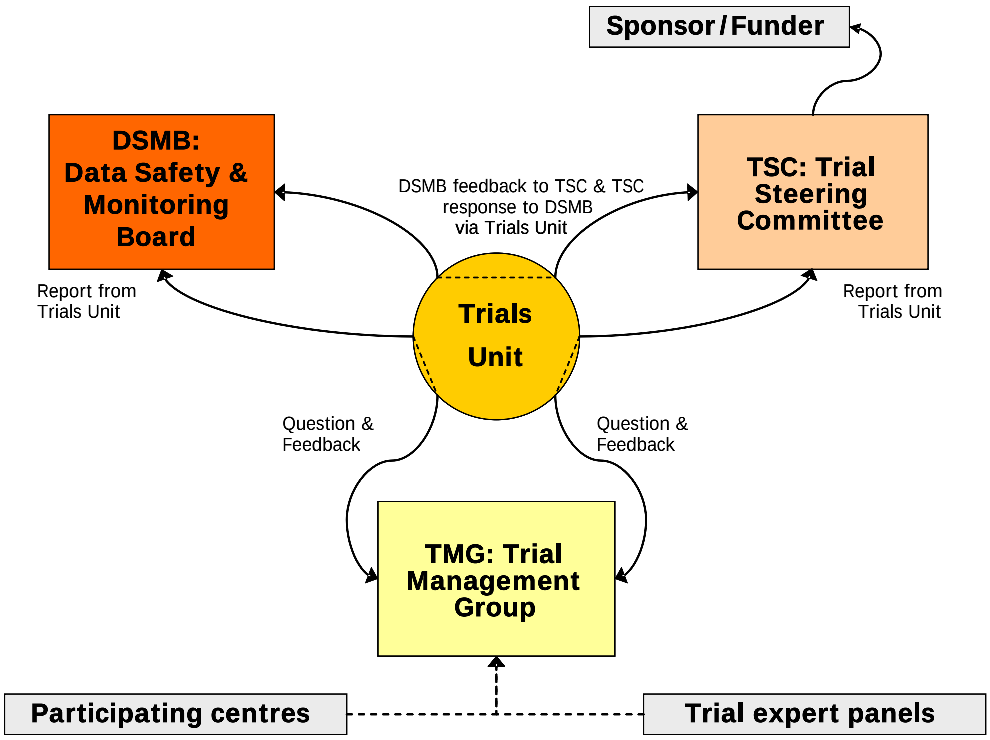

abbreviations:

BSCTU	Brighton and Sussex Clinical Trials Unit
CI	Chief Investigator
EUDRACT	European Union Directive on Randomised Controlled Trials
DSMB	Data and Safety Monitoring Board
ISRCTN	International Standardised Randomised Controlled Trial Number
REC	Research Ethics Committee
PI	Principal Investigator
TM	Trial Manager
TMG	Trial Management Group
TSC	Trial Steering Committee

Appendix 1: Agreement and competing interests form for independent members of the PIBIK Data Safety and Monitoring Board

Please complete the following document and return to: PIBIK Clinical Trial Manager at bsctu@bsms.ac.uk

☐ I have read and understood the PIBIK DSMB charter V2.0 dated 22 Nov 2019

☐ I agree to join the DSMB for this trial as an independent member

☐ I agree to treat all sensitive trial data and discussions confidential

The avoidance of any perception that members of the DSMB may be biased in some fashion is important for the credibility of the advice given by the DSMB and for the integrity of the trial.

Possible competing interests should be disclosed via the trials office. In many cases simple disclosure up front should be sufficient. Table 1 lists potential competing interests:

Table 1: Potential competing interests for independent members

1. Stock ownership in any commercial companies involved.
2. Stock transaction in any commercial company involved (if previously holding stock)
3. Consulting arrangements with the Sponsor
4. Frequent speaking engagements on behalf of the intervention
5. Career tied up in a product or technique assessed by the trial
6. Hands-on participation in the trial
7. Involvement in the running of the trial
8. Emotional involvement in the trial
9. Intellectual conflict (e.g. strong prior belief in the trial's experimental arm)
10. Involvement in regulatory issues relevant to the trial procedures
11. Investment (financial or intellectual) in competing products
12. Involvement in publications

☐ **NO,** I have no competing interests to declare

☐ **YES,** I have competing interests to declare (please detail below)

____

____

____

Name: ______________________________

Signed: ______________________________ Date: _______________

Appendix 2: Suggested report from DSMB to TSC where no recommendations are being made

[Insert date]

To: [Chair of Trial Steering Committee]

and Chair of Trial Management Group

Dear,

The Independent Data and Safety Monitoring Board (DSMB) for the xxxxx trial met on *[meeting date]* to review its progress and interim data. *[List members]* were able to attend the meeting and review the report on the trial progress from the CTU.

We congratulate the trial organisers and collaborators on the progress and conduct of the trial and presentation of the data. The trial question remains important and, on the basis of the data reviewed at this stage, we recommend continuation of the trial according to the current version of the protocol *[specify protocol version number and date]* with no changes.

We shall next review the progress and data *[provide approximate timing]*

Yours sincerely,

[Name of meeting Chair]

Chair of Data and Safety Monitoring Board

On behalf of the DSMB (all members listed below)

DSMB members:

(1) [Insert name and role]

(2) [Insert name and role]

(3) [Insert name and role]

(4) [Insert name and role]

Annexe 3: Summarise changes from previous version

Version	Date	Summary of changes
1.0	13 Sep 2019	N/A
2.0	22 Nov 2019	• Clarification DSMB reports to TSC only and any DSMB recommendations will be communicated via TSC to the Sponsor and TMG<br>• DSMB Chair's email address and title updated<br>• Trial Manager will only be available to take minutes in the open session<br>• Other minor administrative and formatting changes
